# Recombinant production of acidophilic L-arabinose isomerase from *Lentilactobacillus parakefiri* in *Bacillus subtilis*

**DOI:** 10.1186/s12934-025-02900-z

**Published:** 2025-12-22

**Authors:** Nathanael Weber, Sebastian Götz, Jana Senger, Sabine Lutz-Wahl, Lutz Fischer

**Affiliations:** 1 Institute of Food Science and Biotechnology, Department of Biotechnology and Enzyme Science, University of Hohenheim, Garbenstr. 25, 70599 Stuttgart, Germany; 2 Present Address: Institute for Applied Biotechnology, Biberach University of Applied Sciences, Karlstr. 11, 88400 Biberach, Germany

**Keywords:** Bacillus subtilis, Nonsporulating strain, Lentilactobacillus parakefiri, L-arabinose isomerase, CRISPR/Cas9

## Abstract

**Background:**

The monosaccharide D-tagatose is a promising alternative to sucrose because of its similar sweetness and lower glycemic index. A novel L-arabinose isomerase (L-AI) from *Lentilactobacillus parakefiri* DSM 10551 (L-AI-Lp) has been biochemically characterized and used to isomerize D-galactose to D-tagatose in skim milk ultrafiltration permeate at pH 4.5 and 6.5. However, like most L-AIs described in the literature, this enzyme has only been produced recombinantly in *Escherichia coli*. This study aimed to systematically investigate the intracellular recombinant production of L-AI-Lp in *Bacillus subtilis*, which has qualified for a presumption of safety (QPS) designation from the European Food Safety Authority.

**Results:**

The influence of four promoters on L-AI-Lp production in *B. subtilis* 007 was investigated in shake flask cultivations. Among these, the P_AprE_ promoter yielded the highest volumetric L-AI activity of 69.2 ± 7.4 µkat_Gal, 65 °C_/L_Culture_. The production yield was further increased to 147.7 ± 1.0 µkat_Gal, 65 °C_/L_Culture_ by using the nonsporulating, surfactin-deficient strain *B. subtilis* 007 ∆*sfp* ∆*sigF*, which was constructed by deleting *sigF* and *sfp* in *B. subtilis* 007. Furthermore, the influence of pH and dissolved oxygen (DO) on bioreactor cultivations of *B. subtilis* 007 ∆*sfp* ∆*sigF* was analyzed. In bioreactor cultivations, the highest L-AI activity of 88.6 ± 2.4 µkat_Gal, 65 °C_/L_Culture_ was measured under unregulated pH and low oxygen conditions (DO ≤ 5%), representing a 3.2-fold increase compared with previous recombinant production in *E*. *coli*. The L-AI-Lp was subsequently partially purified by heat treatment and precipitation methods, resulting in a 7.8-fold increase in specific activity to 128.2 nkat_Gal, 65 °C_/mg and a yield of 84%.

**Conclusions:**

The L-AI-Lp was recombinantly produced for the first time in a microbial species with QPS status using the nonsporulating and surfactin-deficient strain *B. subtilis* 007 ∆*sfp* ∆*sigF*. The L-AI-Lp was subsequently partially purified via nonchromatographic methods, providing a basis for a low-cost downstream process. These results represent an important step toward potential industrial application of L-AI-Lp and highlight the potential of *B. subtilis* 007 ∆*sfp* ∆*sigF* as an expression host for the recombinant production of L-AIs compared with previously used hosts from the order *Lactobacillales*.

**Supplementary Information:**

The online version contains supplementary material available at 10.1186/s12934-025-02900-z.

## Background

L-Arabinose isomerase (L-AI, EC 5.3.1.4) is an enzyme in the bacterial L-arabinose catabolic pathway that catalyzes the aldose-ketose isomerization of L-arabinose to L-ribulose [1]. In addition to its native substrate, most characterized L-AIs also catalyze the isomerization of D-galactose to D-tagatose due to structural similarities between these substrates [2, 3]. D-tagatose, a monosaccharide with a relative sweetness of 92 (compared to 100 for sucrose) in 10% (w/v) solutions and a relatively low glycemic index of 3 (compared to 65 for sucrose), has been identified as a potential alternative to sucrose [4, 5]. The U.S. Food and Drug Administration and the European Food Safety Authority have calculated its physiological caloric value to be 6 kJ/g and 13 kJ/g, respectively, both of which are lower than that of sucrose (17 kJ/g) [6, 7].

A novel L-AI from *Lentilactobacillus parakefiri* DSM 10551 (L-AI-Lp) was biochemically characterized for the isomerization of D-galactose to D-tagatose in a recent study [8]. The L-AI-Lp had a relative activity of ≥ 70% across a wide pH range of pH 4.0–9.0 and isomerized 45% of D-galactose in sodium acetate buffer at pH 4.5 [8]. Furthermore, a similar isomerization of 50% was observed in skim milk ultrafiltration permeate at pH 4.5. The L-AI-Lp, in combination with a commercial *β*-galactosidase (EC 3.2.1.23) preparation, converted 100 g/L lactose to around 23 g/L D-tagatose [9]. To date, the L-AI-Lp is the only L-AI reported to isomerize D-galactose to D-tagatose in a dairy byproduct at pH ≤ 5.0 [9]. Furthermore, the L-AI-Lp exhibited relatively high storage stability at 4 °C and − 20 °C in buffer containing 1 mM CoCl_2_. The L-AI retained 75 ± 4% and 97 ± 4% of its activity after 6 months at 4 °C and − 20 °C, respectively [9]. Cobalt (Co^2+^) ions were added because the L-AI-Lp showed maximal activity in their presence [8]. In contrast, glycerol, a widely used protein-stabilizing cosolvent that reduces aggregation in aqueous solutions, has not yet been tested for L-AI-Lp [10].

Similar to most L-AIs described in the literature, L-AI-Lp has only been produced in *Escherichia coli*, yielding a volumetric (vol.) activity of 27.72 µkat_Gal 65 °C_/L_Culture_ during bioreactor cultivation [8, 11, 12]. However, some studies have focused on the intracellular production of L-AIs in prokaryotes with a qualified presumption of safety (QPS), as defined by the European Food Safety Authority [13–16]. Accordingly, expression hosts such as *Lactococcus lactis*, *Lactobacillus plantarum*, and *Bacillus subtilis* have been used [13–15].

*B. subtilis* is a Gram-positive soil bacterium that has been extensively studied as a microbial cell factory for the production of heterologous enzymes [17]. Furthermore, the ability of *B*. *subtilis* to reach high cell densities has been demonstrated by Klausmann et al. [18]. In this study, *B. subtilis* was grown to a dry cell weight (DCW) of up to 88 g/L during fed-batch cultivation for surfactin production [18].

The well-studied strain *B. subtilis* 168 has been used for the production of various enzymes, including *β*-glucuronidase, aspartase, and transglutaminase [19–21]. Furthermore, *B*. *subtilis* 168 has an endogenous L-AI that isomerizes L-arabinose but not D-galactose [22]. Although undomesticated *B*. *subtilis* strains are often less amenable to genetic transformation, the strain *B. subtilis* 007 has been used as an expression host for the recombinant production of *β*-galactosidase, cellobiose-2-epimerase, and *β*-glucosidase [23].

Several studies have optimized cultivation parameters, such as pH and dissolved oxygen (DO), to improve recombinant enzyme production in *B*. *subtilis*, thereby supporting systematic process development [24, 25]. However, sporulation and excessive foaming may occur during heterologous enzyme production in *B. subtilis* as a result of nutrient limitation and the synthesis of biosurfactants (e.g., surfactin), respectively [26, 27]. These can lead to contamination risks and reduced enzyme yields [28, 29]. Consequently, studies have focused on engineering of nonsporulating and surfactin-deficient strains by deleting sporulation- and surfactin-related genes [30, 31].

The objective of the present study was to investigate the potential of *B. subtilis* as an expression host for the intracellular production of L-AI-Lp. The influence of four promoters (P_AprE_, P_SecA_, P_SacB_, and P_ManP_) on L-AI production was therefore compared using the strain *B*. *subtilis* 007. Furthermore, a nonsporulating and surfactin-deficient strain (*B. subtilis* 007 ∆*sfp* ∆*sigF*) was constructed using the CRISPR/Cas9 system by deleting *sfp* (encoding the phosphopantetheinyl transferase Sfp, EC 2.7.8.7) and *sigF* (encoding the RNA polymerase sigma factor SigF). This strain was then compared with *B. subtilis* 007 and *B. subtilis* 168 as expression hosts for L-AI-Lp production in shake flask cultivations. Subsequent bioreactor cultivations were done to analyze the influences of pH and DO and to evaluate the potential of *B. subtilis* 007 ∆*sfp* ∆*sigF* to produce L-AI-Lp. Finally, the L-AI was partially purified using nonchromatographic methods, and the influence of Co^2+^ ions and glycerol on the storage stability of L-AI-Lp was investigated.

## Methods

### Chemicals and enzymes

All chemicals used in this study were of analytical grade and obtained from Carl Roth GmbH (Karlsruhe, Germany), Fisher Scientific (Hampton, USA), or Sigma-Aldrich (St. Louis, USA), unless otherwise stated. Yeast extract and soy peptone were purchased from Carl Roth GmbH (Karlsruhe, Germany). The primers were synthesized by Biomers (Ulm, Germany). Q5^®^ high-fidelity DNA polymerase, shrimp alkaline phosphatase, and the restriction enzymes *Bsm*BI, *Eco*RI, *Sfi*I, *Spe*I, and *Xho*I were obtained from New England Biolabs GmbH (Frankfurt, Germany). T4 DNA ligase was purchased from Thermo Fisher Scientific (Hampton, USA). Lysozyme from chicken egg white was obtained from SERVA Electrophoresis GmbH (Heidelberg, Germany).

### Bacterial strains and media

*E*. *coli* XL1 was cultivated in Luria–Bertani (LB) medium supplemented with 100 µg/mL ampicillin at 37 °C. The *B. subtilis* strain 007 (DSM 118688) was obtained from Senger et al. [23], and *B. subtilis* 168 (DSM 402) was purchased from the Leibniz Institute DSMZ (Braunschweig, Germany). Both *B. subtilis* strains and *B. subtilis* 007 ∆*sfp* ∆*sigF* (constructed in this study; see below) were grown in starvation medium 1 and starvation medium 2 for transformation, as described previously [32]. Starvation medium 1 consisted of (NH_4_)_2_SO_4_ (2 g/L), K_2_HPO_4_ (14 g/L), KH_2_PO_4_ (6 g/L), Na_3_Citrate (0.7 g/L), glucose (5 g/L), MgSO_4_·7H_2_O (0.2 g/L), yeast extract (2 g/L), and soy peptone (0.25 g/L). Starvation medium 2 was composed of (NH_4_)_2_SO_4_ (2 g/L), K_2_HPO_4_ (14 g/L), KH_2_PO_4_ (6 g/L), Na_3_Citrate (0.7 g/L), glucose (5 g/L), MgSO_4_·7H_2_O (0.8 g/L), yeast extract (1 g/L), soy peptone (0.1 g/L), and CaCl_2_ (0.5 g/L). *B. subtilis* strains were cultivated in seed medium (sucrose 40 g/L; soy peptone 30 g/L; KH_2_PO_4_ 6 g/L; MgCl_2_·6H_2_O 2.04 g/L) and fermentation medium (sucrose 70 g/L; soy peptone 50 g/L; KH_2_PO_4_ 5 g/L; MgCl_2_·6H_2_O 3.06 g/L), as described by Zhang et al. for the recombinant production of L-AI-Lp [24]. However, mannose (73.69 g/L) was used instead of sucrose as the carbon source for the *B. subtilis* strain *Bs*007-Ma, which has the mannose-inducible promoter P_ManP_ (see below).

### Construction of expression plasmids

The list of primers, plasmids, and recombinant *B. subtilis* strains constructed and used in the present study is provided in the Additional file 1: Tables S1, S2, and S3, respectively. The expression plasmids for the production of L-AI-Lp (National Center for Biotechnology Information protein_ID: KRL70137.1) were constructed based on a modular expression cassette previously integrated into the pLF shuttle vector [33]. The cassette contains restriction sites for exchanging the promoter and the gene of interest via restriction digestion and ligation. The vector and the insert were digested with appropriate restriction enzymes, and the vector was subsequently incubated with shrimp alkaline phosphatase to prevent self-ligation. After agarose gel electrophoresis, the samples were excised from 1% (w/v) agarose gels and purified using the GeneJET Gel Extraction Kit (Thermo Fisher Scientific, Hampton, USA). Ligation of the insert and vector was done using T4 DNA ligase at a molar ratio of 3:1 (insert: vector) for 1 h at room temperature. The ligation product was transformed into *E*. *coli* XL1 by heat shock [34], and plasmids were isolated from single colonies using the GeneJET Plasmid-Miniprep Kit (Thermo Fisher Scientific, Hampton, USA). Cloning was confirmed by sequencing (Eurofins Genomics, Ebersberg, Germany).

The following amplifications were done by polymerase chain reaction (PCR) using Q5 DNA polymerase. The resulting PCR products were purified using the DNA Clean & Concentrator Kit (Zymo Research, Orange, USA).

The native *L-AI-Lp* gene was amplified from the genomic DNA of *Lentilactobacillus parakefiri* DSM 10551 (National Center for Biotechnology Information GenBank: AZEN01000120.1), which was purchased from the Leibniz Institute DSMZ (Braunschweig, Germany). The amplification was done with primers P1 and P2. The PCR product was cloned into the plasmid pLF_P_AprE__AprE, provided by Senger et al. [33], via *Spe*I/*Xho*I restriction digestion, resulting in the expression plasmid pLF_P_AprE__L-AI-Lp (Additional file 1: Figure S1).

The pLF_P_SacB__L-AI-Lp plasmid was constructed by replacing the P_AprE_ promoter in pLF_P_AprE__L-AI-Lp with the P_SacB_ promoter. The P_SacB_ promoter was amplified from *B. subtilis* 007 genomic DNA using primers P3 and P4. Genomic DNA was extracted using the GeneJET Genomic DNA Purification Kit (Thermo Fisher Scientific, Hampton, USA). Both the amplified promoter fragment and the plasmid pLF_P_AprE__L-AI-Lp were digested with *Eco*RI and *Spe*I, resulting in the final expression plasmid pLF_P_SacB__L-AI-Lp after ligation.

The promoter sequence P_AprE_ from the original plasmid pLF_P_AprE__L-AI-Lp was exchanged with the promoter P_ManP_ to construct the expression plasmid pLF_P_ManP__L-AI-Lp. The primers P5 and P6 were used to amplify the promoter P_ManP_ from the plasmid pJOE8999, which was derived from a previous study [35]. The resulting fragment was then cloned into pLF_P_AprE__L-AI-Lp via *Eco*RI and *Spe*I restriction digestion to obtain the plasmid pLF_P_ManP__L-AI-Lp.

The P_SecA_ promoter was amplified from *B. subtilis* 168 genomic DNA with primers P7 and P8 to construct the plasmid pLF_P_SecA__L-AI-Lp. Genomic DNA was extracted as described above. The amplified P_SecA_ promoter fragment was subsequently cloned into the pLF vector. Due to the presence of a *Spe*I site within the P_SecA_ promoter sequence, the amplified promoter fragment and plasmid pLF_P_AprE__AprE were digested with *Eco*RI/*Bsm*BI and *Eco*RI/*Spe*I, respectively. The resulting plasmid pLF_P_SecA__AprE was amplified with the primers P9 and P10. In parallel, the *L-AI-Lp* gene was amplified from *Lentilactobacillus parakefiri* DSM 10551 genomic DNA with primers P11 and P12 to generate homologous overhangs. The Gibson Assembly Cloning Kit (New England BioLabs GmbH, Frankfurt, Germany) was used to ligate both DNA fragments, resulting in the final expression plasmid pLF_P_SecA__L-AI-Lp.

### Construction of *B. subtilis* 007 ∆*sfp* ∆*sigF*

The CRISPR/Cas9-mediated deletions were done based on plasmid pJOE8999 [35]. The primers and plasmids used are listed in Tables S1 and S2 (Additional File 1). The plasmids pJOE8999_sgSigF and pJOE8999_sgSfp were constructed previously and contain the sgRNA targeting the deletion of *sigF* and *sfp*, respectively [36]. The repair template was inserted into pJOE8999_sgSigF and pJOE8999_sgSfp, generating pJOE8999_DsigF and pJOE8999_Dsfp. Therefore, 800–900 bp flanking regions of *sigF* or *sfp* were amplified by PCR with 5’ *Sfi*I overhangs using the Q5 DNA polymerase. Primer pairs P13/P14 and P15/P16 were used to amplify the *sigF* flanks, while P17/P18 and P19/P20 were used for the *sfp* flanks. Genomic DNA from *B. subtilis* 007, which was isolated using the GeneJET genomic DNA Purification Kit (Thermo Fisher Scientific, Hampton, USA), served as a template for PCR. The PCR products were purified using the DNA Clean & Concentrator Kit (Zymo Research, Orange, USA) and digested with *Sfi*I at 50 °C for 3–14 h. Plasmids pJOE8999_sgSigF and pJOE8999_sgSfp were similarly *Sfi*I-digested and additionally treated with shrimp alkaline phosphatase. After digestion, the DNA fragments were separated via 1% (w/v) agarose gel electrophoresis and extracted using the GeneJET Gel Extraction Kit (Thermo Fisher Scientific, Hampton, USA). The digested plasmids and PCR products were ligated with 100 ng of vector, 20 ng of upstream flank, and 20 ng of downstream flank using T4 DNA ligase. The ligation samples were used for heat shock transformation of *E. coli* XL1, which was plated on LB agar plates with 50 µg/mL kanamycin. Plasmids were isolated from single colonies using the GeneJET Plasmid Miniprep Kit (Thermo Fisher Scientific, Hampton, USA). The correct integration of the repair template was confirmed by digestion and sequencing (Eurofins Genomics, Ebersberg, Germany).

Deletions of *sigF* and *sfp* were done as described previously [36]. *B. subtilis* 007 was transformed with the plasmids pJOE8999_DsigF or pJOE8999_Dsfp and plated on LB plates with 0.5% (w/v) mannose and 5 µg/mL kanamycin. The plates were then incubated either overnight at 37 °C or for two days at 30 °C. For plasmid curing, single colonies were streaked on LB plates and incubated at 50 °C for 9–15 h. The single clones were then streaked on LB plates again and incubated overnight at 42 °C. Clones that lost the plasmid did not grow on LB plates with kanamycin. Gene deletions were verified by colony PCR using the Taq DNA Polymerase Kit (TaKaRa Bio Inc., Shiga, Japan). For genomic DNA extraction, single colonies were inoculated into 50 µL of 20 mM NaOH. After heating the sample for 10 min at 95 °C, an amount of 2 µL of the supernatant was used as a template for colony PCR.

### Transformation of *B. subtilis* strains

Plasmid DNA was amplified using the Cytiva Illustra™ Templiphi™ Kit (Fisher Scientific, Hampton, USA), according to the manufacturer’s instructions, and stored at 5 °C until transformation into *B. subtilis*. Transformation was done with naturally competent *B. subtilis* cells (007, 007 ∆*sfp* ∆*sigF*, 168), according to the protocol described by Vojcic et al., with minor modifications [32]. In contrast to the original method, no histidine was added after the culture volume was doubled with starvation medium 2. Furthermore, transformation was done using approximately 2000 ng of plasmid DNA, and transformed cells were plated on LB agar plates containing neomycin (7.5 µg/mL). The cells were then incubated at 37 °C for approximately 16 h.

### Shake flask cultivation of the recombinant *B. subtilis* strains

Precultures were prepared by inoculating 15 mL of seed medium containing 7.5 µg/mL neomycin with recombinant *B. subtilis* cells and incubating at 37 °C and 180 rpm for 9 h in 100 mL baffled shake flasks. The precultures were subsequently used to inoculate 150 mL fermentation medium in 1 L baffled shake flasks to an optical density at 600 nm (OD_600_) of 0.05. The main cultures were then incubated at 30 °C and 110 rpm for 67 h. Samples were taken at predefined and similar time points during all shake flask cultivations to determine the OD_600_, pH, protein concentration, and intracellular L-AI activity (as described below).

### Bioreactor cultivations of *Bs*007∆*ss*-Ap

Two separate batch bioreactor cultivations of *B. subtilis* 007 ∆*sfp* ∆*sigF* with the plasmid pLF_P_AprE__L-AI-Lp (*Bs*007∆*ss*-Ap) were done in 1 L Multifors bioreactor systems (Infors HT, Bottmingen, Switzerland), each containing 0.8 L of fermentation medium. Freshly transformed *Bs*007∆*ss*-Ap cells were used to inoculate 15 mL of seed medium into 100 mL baffled shake flasks (preculture I), which were then incubated at 37 °C and 180 rpm for 9 h. Subsequently, 10 mL of preculture I was transferred to 190 mL of seed medium in 1 L baffled shake flasks (preculture II) and incubated at 37 °C and 110 rpm for 14–16 h. Finally, 80 mL of preculture II was used to inoculate 720 mL of fermentation medium in the bioreactor.

The temperature was maintained at 30 °C, and the aeration rate was set to 1.0 vvm in both cultivations. One cultivation was done for 65 h under unregulated pH conditions, with DO maintained at ≤ 5% air saturation. The other cultivation was done for 30 h at a controlled pH of 7.0 (maintained by the addition of 2 M NaOH and 2 M H_3_PO_4_), with DO > 30% by adjusting the stirrer speed. The pH and DO were measured in both cultivations using the 405-DPAS-SC-K8S pH electrode and an InPro 6900 oxygen sensor (Mettler Toledo, Columbus, USA), respectively. Antifoam 204 (Sigma-Aldrich, St. Louis, USA) was added manually when needed.

At the end of cultivation, the cells were harvested by centrifugation at 8000 × g for 30 min at 4 °C. The cell pellets were then washed with 0.9% (w/v) NaCl, centrifuged again, and stored at -80 °C until further use.

In addition, samples were taken at defined time points during cultivation to determine the OD_600_, DCW, protein concentration, and intracellular L-AI activity (as described below). The intracellular proteins of *Bs*007∆*ss*-Ap were further analyzed by sodium dodecyl sulfate‒polyacrylamide gel electrophoresis (SDS‒PAGE), as described by Weber et al. [8]. The glucose and sucrose concentrations in the culture supernatant were quantified using the Enzytec™ Liquid Sucrose/D-Glucose Kit, according to the manufacturer’s instructions (R-Biopharm AG, Darmstadt, Germany).

### Sample preparation and determination of protein concentration

Samples from the shake flask and bioreactor cultivations were taken and centrifuged at 8000 × g and 4 °C for 10 min. The resulting cell pellets were washed by resuspension in 0.9% (w/v) NaCl, followed by a second centrifugation step under the same conditions. A 30% (w/v) cell suspension was subsequently prepared in 2-(*N*-morpholino)ethanesulfonic acid (MES) buffer (25 mM, pH 6.5) containing 1 mM CoCl_2_, 1 mg/mL lysozyme, and 1 mM phenylmethylsulfonyl fluoride. The cell suspension was incubated for 1 h at room temperature to lyse the cells enzymatically. The cell debris was removed by centrifugation at 13,000 × g for 15 min at 4 °C. The resulting supernatant (cell-free extract) was used for the determination of L-AI activity and protein concentration. Protein quantification was done using the Bradford method with bovine serum albumin as the standard [37].

### Standard L-AI activity assay

The L-AI activity was determined using D-galactose as a substrate, as described previously by Weber et al., with minor modifications [8]. Specifically, the L-AI solution and the substrate solution (600 mM D-galactose dissolved in 25 mM MES pH 6.5 with 1 mM CoCl_2_) were preincubated separately at 65 °C. The isomerization was started by adding 100 µL of the L-AI solution to 100 µL of the D-galactose solution. The reaction was stopped after 5–10 min by the addition of 200 µL of HCl (1 M). Sample preparation and quantification of D-tagatose were done as described by Weber et al. [8]. One katal was defined as the amount of enzyme that converts 1 mol D-galactose to D-tagatose per second.

### Partial purification of L-AI-Lp

The L-AI-Lp was partially purified by a combination of heat treatment, polyethyleneimine precipitation, and ammonium sulfate precipitation. The harvested cells from the bioreactor cultivation of *Bs*007∆*ss*-Ap with unregulated pH and DO ≤ 5% were disrupted by lysozyme. Therefore, 20.5 g of wet cell biomass was thawed on ice and resuspended to a 30% (w/v) suspension in MES buffer (100 mM, pH 6.0) supplemented with 1 mM CoCl_2_, 1 mg/mL lysozyme, and 1 mM phenylmethylsulfonyl fluoride. The suspension was incubated at room temperature for 1 h to lyse the cells. Subsequently, the cell debris was removed by centrifugation (13,000 × g, 20 min, 4 °C), and the resulting supernatant was heated at 65 °C for 45 min. The denatured proteins were then removed by centrifugation (13,000 × g, 15 min, 4 °C), and the resulting supernatant was used for polyethyleneimine precipitation. A 10% (w/v) polyethyleneimine solution (pH 7.1) was added dropwise to the supernatant to a final concentration of 0.1% (w/v) while stirring on ice. After 1 h of equilibration, the mixture was centrifuged (13,000 × g, 15 min, 4 °C), and the supernatant was used for fractionated ammonium sulfate precipitation. A 4 M ammonium sulfate solution was added dropwise to the supernatant to obtain an ammonium sulfate saturation of 60%, followed by stirring on ice for 1 h. The mixture was centrifuged (13,000 × g, 15 min, 4 °C), and the supernatant was used for a second consecutive precipitation by increasing the ammonium sulfate saturation to 85%. After 1 h of equilibration on ice, the mixture was centrifuged again (13,000 × g, 15 min, 4 °C). The resulting pellet containing L-AI-Lp was resuspended in MES buffer (25 mM, pH 6.5) supplemented with 1 mM CoCl_2_.

Samples were taken after cell disruption and each purification step to determine the L-AI activity and protein concentration. The samples were desalted into MES buffer (25 mM, pH 6.5, 1 mM CoCl_2_) using PD10 columns (GE Healthcare, Düsseldorf, Germany). The partial purification products were further analyzed by SDS‒PAGE, as described by Weber et al. [8].

### Influence of cobalt ions and glycerol on the storage stability of L-AI-Lp

The influence of Co^2+^ ions and glycerol on the storage stability of partially purified L-AI-Lp was investigated at 4 °C and − 20 °C for 6 months (1 month = 29 days). The L-AI-Lp was desalted using PD10 columns (GE Healthcare, Düsseldorf, Germany) into 25 mM MES buffer (pH 6.5) without additives, with 1 mM CoCl_2_, or with 40% (v/v) glycerol. These conditions were tested separately and were not combined.

Aliquots of L-AI-Lp (2.3 g/L) in MES buffer without additives (286.9 ± 3.3 nkat_Gal, 65 °C_/mL), with CoCl_2_ (278.0 ± 16.6 nkat_Gal, 65 °C_/mL), or with glycerol (226.0 ± 13.0 nkat_Gal, 65 °C_/mL) were stored at 4 °C and − 20 °C. The residual L-AI activity was determined after 1, 2, 3, and 6 months using the standard L-AI activity assay.

### Data analysis

All experiments were done with at least two biological replicates, each with three independent measurements, and were evaluated by determining the standard deviation with Microsoft Excel (Microsoft, Redmond, USA). The data are presented as mean values with standard deviations. Statistical analyses were done using ANOVA and t-test in MATLAB (MathWorks, Natick, USA).

## Results

### Influence of promoters on the recombinant production of L-AI-Lp

Expression plasmids for the intracellular production of L-AI-Lp in *B*. *subtilis* were constructed using a modular cassette previously integrated into the shuttle vector pLF [33]. The native *L-AI-Lp* gene was cloned into the cassette of the plasmid pLF_P_AprE__AprE from Senger et al. [33], resulting in the plasmid pLF_P_AprE__L-AI-Lp (Additional file 1: Table S2). The plasmid enabled the expression of L-AI-Lp under the control of the P_AprE_ promoter. The influence of different promoters on L-AI-Lp production was investigated by replacing the P_AprE_ promoter with the promoters P_SecA_, P_SacB_, and P_ManP_ [38–40], resulting in the plasmids pLF_P_SecA__L-AI-Lp, pLF_P_SacB__L-AI-Lp, and pLF_P_ManP__L-AI-Lp (Additional file 1: Table S2). *B. subtilis* 007 was individually transformed with these plasmids, including pLF_P_AprE__L-AI-Lp, resulting in four corresponding strains: *Bs*007-Ap, *Bs*007-Se, *Bs*007-Sa, and *Bs*007-Ma (Additional file 1: Table S3).

**Fig. 1:**
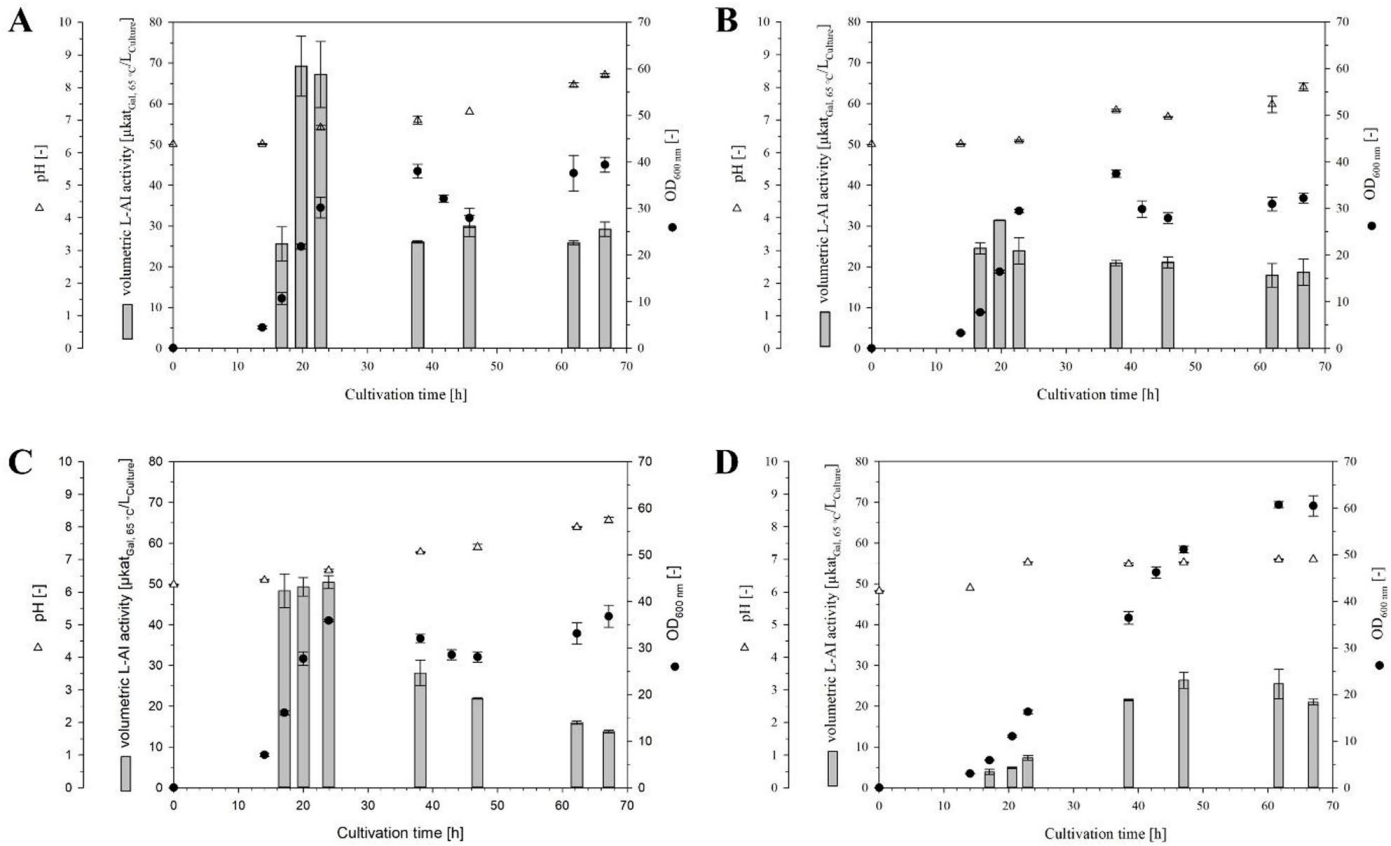
Shake flask cultivations for intracellular production of L-AI-Lp in *B. subtilis* 007 using different promoters. The batch cultivations of *Bs*007-Ap (**A**), *Bs*007-Se (**B**), *Bs*007-Sa (**C**), and *Bs*007-Ma (**D**) were done with a working volume of 150 mL at 30 °C

The recombinant strains *Bs*007-Ap, *Bs*007-Se, and *Bs*007-Sa were cultivated in 150 mL of fermentation medium with sucrose as the carbon source [24]. By contrast, sucrose was replaced by mannose for *Bs*007-Ma because the P_ManP_ promoter is induced by mannose [41]. The strains *Bs*007-Ap, *Bs*007-Se, and *Bs*007-Sa showed similar growth behavior, with the OD_600_ increasing to approximately 37 within 24–38 h, followed by a decrease to ≈ 28 after 46 h. Subsequently, the OD_600_ increased slightly to 32–39 after 67 h (Fig. 1A–C). The fluctuations in the OD_600_ observed during sucrose-based cultivations may be attributed to cell lysis and sporulation. However, these changes in the OD_600_ over time were not observed in the cultivation of *Bs*007-Ma when mannose was used as the carbon source. The OD_600_ increased continuously, reaching a maximum of 62 after 67 h (Fig. 1D). The lower OD_600_ in sucrose-based cultivations may result from an increase in viscosity of the fermentation broth associated with the formation of exopolysaccharides (e.g., levan). Levan can be synthesized in sucrose-rich cultivation media by levansucrase (EC 2.4.1.10) [42]. The pH of the fermentation broths shifted from an initial pH ≈ 6.2 to a final pH ≈ 7.0–8.4 during all cultivations (Fig. 1A–D). This pH increase has previously been described for other *B. subtilis* strains cultivated in complex soy media [24, 33], and may have been enhanced by cell lysis. Among the recombinant *B*. *subtilis* 007 strains, the highest vol. and OD_600_-normalized L-AI activities were measured for *Bs*007-Ap after ≈ 20 h, with 69.2 ± 7.4 µkat_Gal, 65 °C_/L_Culture_ and 3.2 ± 0.3 µkat_Gal, 65 °C_/L_Culture_/OD_600_, respectively (Table 1). Thereafter, the vol. activity decreased, probably due to proteolysis of L-AI-Lp, cell lysis, or both.

**Table 1:** Comparison of intracellular L-AI-Lp production in *B. subtilis* 007 using different promoters

Recombinant strain	Bs007-Ap	Bs007-Se	Bs007-Sa	Bs007-Ma
Promoter	P_AprE_	P_SecA_	P_SacB_	P_ManP_
Carbon source	Sucrose	Sucrose	Sucrose	Mannose
Highest vol. L-AI activity [µkat_Gal, 65 °C_/L_Culture_]	69.2 ± 7.4	31.4 ± 0.1	50.5 ± 1.6	26.4 ± 2.0
Vol. L-AI activity normalized to OD_600_ [µkat_Gal, 65 °C_/L_Culture_/OD_600_]^a^	3.2 ± 0.3	1.9 ± 0.1	1.4 ± 0.1	0.5 ± 0.1

The batch cultivations were done with a working volume of 150 mL at 30 °C. ^a^Calculated with the highest vol. L-AI activity

### Comparison of different *B. subtilis* strains to produce L-AI-Lp

Sporulation and excessive foaming during cultivation are critical for industrial enzyme production because they can lead to incomplete sterilization and reduced product yield [28, 43]. Therefore, the genes *sfp* and *sigF* of *B. subtilis* 007, which are associated with surfactin production [44] and sporulation [45], respectively, were deleted using CRISPR/Cas9 to generate *B. subtilis* 007 ∆*sfp* ∆*sigF.* This strain, along with the widely used strain *B. subtilis* 168 [19–21], was individually transformed with the expression plasmid pLF_P_AprE__L-AI-Lp, resulting in the recombinant strains *Bs*007∆*ss-*Ap and *Bs*168-Ap, respectively (Additional file 1: Table S3). The expression plasmid pLF_P_AprE__L-AI-Lp was selected based on the highest L-AI activities observed during the shake flask cultivation of the strain *Bs*007-Ap. *B. subtilis* 168 does not produce surfactin due to a frameshift mutation in the *sfp* gene [46], but produces endospores in response to stress [47], unlike *B. subtilis* 007 ∆*sfp* ∆*sigF*.

**Fig. 2:**
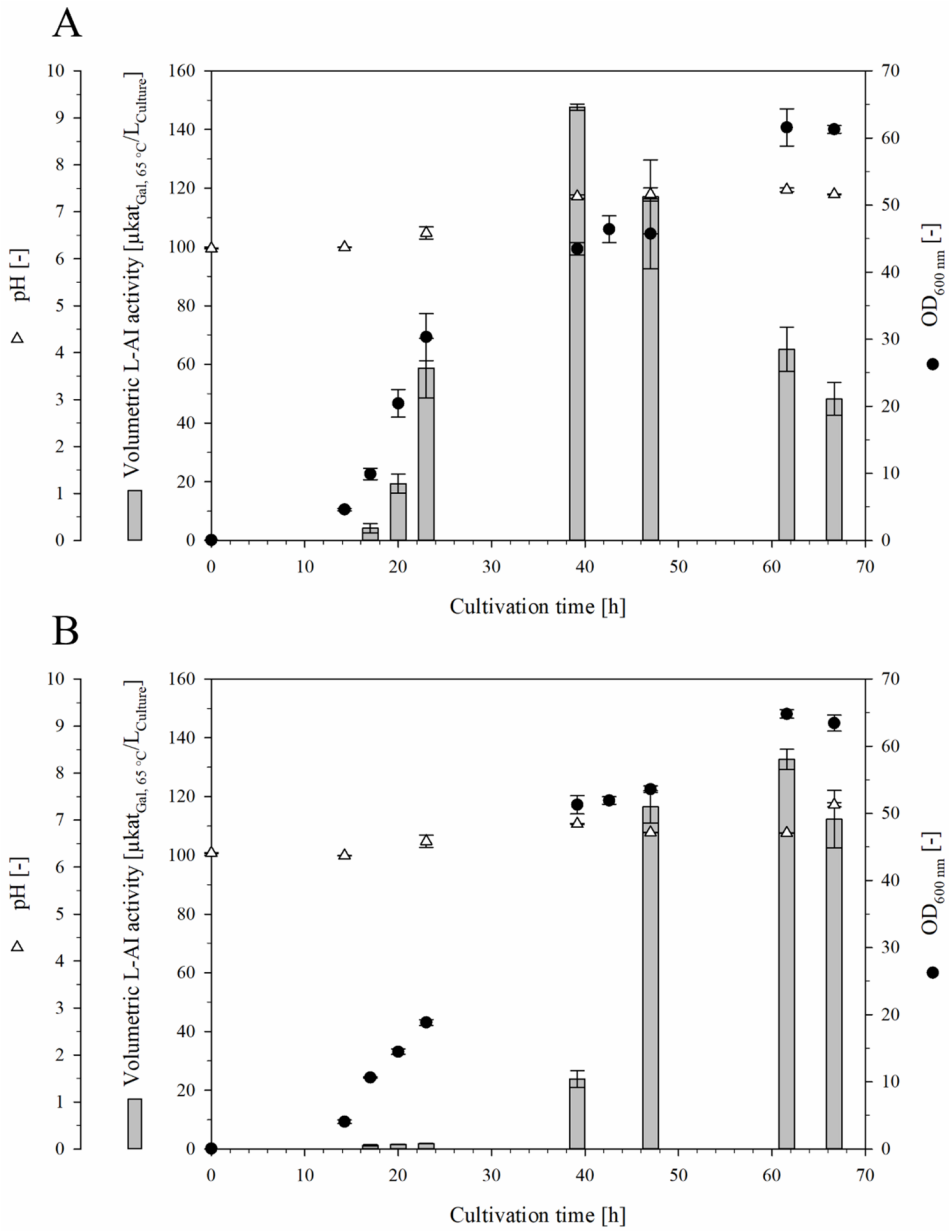
Shake flask cultivations of *Bs*007∆*ss*-Ap (**A**) and *Bs*168-Ap (**B**) for the intracellular production of L-AI-Lp. The batch cultivations were done with a working volume of 150 mL at 30 °C

No differences in colony morphology were observed between the wild-type strain *Bs*007-Ap and *Bs*007∆*ss*-Ap on LB agar. After transformation, the strains *Bs*007∆*ss*-Ap and *Bs*168-Ap were cultivated individually in 150 mL of fermentation medium at 30 °C for 67 h. Both strains showed similar cell growth, with OD_600_ increasing until 62 h and reaching maxima of 62 (*Bs*007∆*ss*-Ap) and 65 (*Bs*168-Ap) (Fig. 2). The pH of the fermentation broths shifted from pH 6.2 to 7.3–7.4 after 67 h. During the cultivation of *Bs*007∆*ss*-Ap, the vol. activity increased and reached the highest measured value of 147.7 ± 1.0 µkat_Gal, 65 °C_/L_Culture_ after 39 h, corresponding to an OD_600_-normalized L-AI activity of 3.4 ± 0.1 µkat_Gal, 65 °C_/L_Culture_/OD_600_. These activities were significantly higher than those observed for *Bs*168-Ap (*p* < 0.001). *Bs*168-Ap reached a vol. activity of 132.7 ± 3.4 µkat_Gal, 65 °C_/L_Culture_ and an OD_600_-normalized activity of 2.1 ± 0.1 µkat_Gal, 65 °C_/L_Culture_/OD_600_ after 62 h. Furthermore, the vol. activity in the cultivation of *Bs*007∆*ss*-Ap was approximately 2-fold higher compared to *Bs*007-Ap, although the OD_600_-normalized L-AI activities were similar between the two strains (Fig. 1A; Table 1). Therefore, the 1.6-fold higher OD_600_ in the cultivation of *Bs*007∆*ss*-Ap was the reason for its higher vol. L-AI activity. The deletion of *sigF* is a possible explanation for the higher OD_600_ because it blocked sporulation and prolonged vegetative growth. Another explanation could be the deletion of *sfp*, which prevented the production of surfactin and probably explains the higher OD_600_ of *Bs*168-Ap compared with *Bs*007-Ap.

Although *B*. *subtilis* 007 has a putative L-AI (National Center for Biotechnology Information GenBank: MGD8065212.1) and *B*. *subtilis* 168 has an endogenous L-AI [22], no intracellular L-AI activity was detected for the negative control strains *Bs*007∆*ss-*nc and *Bs*168-nc (Additional file 1: Figure S2, S3). This confirms that the L-AI activity measured can be attributed to L-AI-Lp.

### Investigation of the bioreactor cultivations of *Bs*007∆*ss*-Ap

The *Bs*007∆*ss*-Ap strain presented the highest vol. L-AI activity of 147.7 ± 1.0 µkat_Gal, 65 °C_/L_Culture_ in the shake flask cultivations. Therefore, this strain was selected for cultivation in 1 L bioreactors with a working volume of 0.8 L. In contrast to shake flask cultivations, the pH was maintained at pH 7.0, and the DO was kept above 30%. In addition, the glucose and sucrose concentrations in the culture supernatant were quantified.

**Fig. 3:**
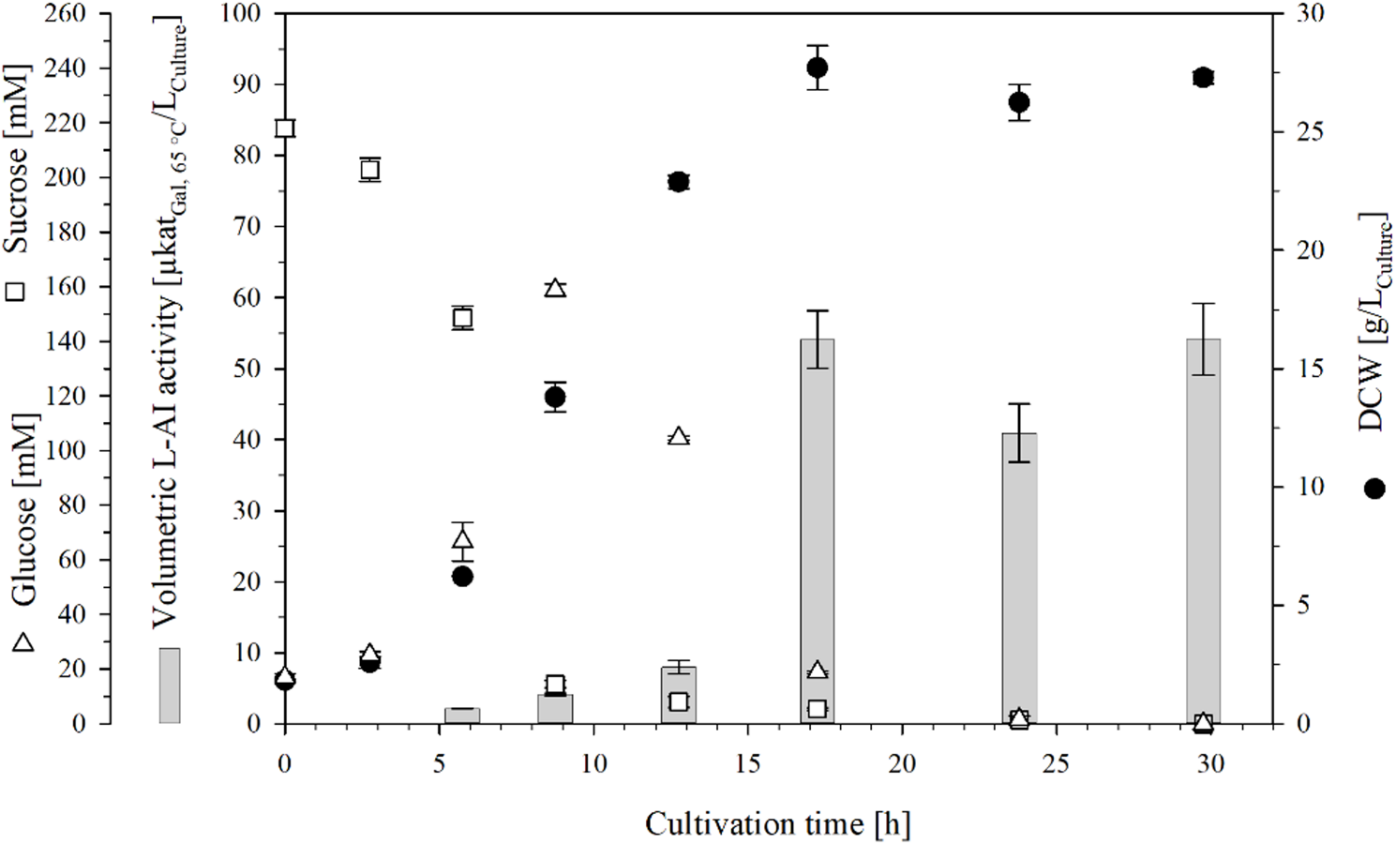
Bioreactor cultivation of *Bs*007∆*ss*-Ap for the intracellular production of L-AI-Lp. The batch cultivation was done with a working volume of 0.8 L at 30 °C, pH 7, and DO > 30%

The sucrose concentration in the culture supernatant decreased continuously during cultivation, while the glucose concentration increased simultaneously to a maximum of 153 mM by the mid-exponential phase (≈ 9 h) (Fig. 3). The glucose concentration subsequently decreased to 18 mM after 17 h and was below the limit of quantification at the end of cultivation. This trend is probably due to the extracellular levansucrase, which hydrolyzes sucrose to release glucose and transfers fructosyl residues to the non-reducing end of a levan chain [48]. In addition, *B*. *subtilis* can take up sucrose as sucrose-6-phosphate, after which it is hydrolyzed by SacA (sucrose-6-phosphate hydrolase, EC 3.2.1.26) intracellularly to glucose-6-phosphate and fructose [49, 50]. However, the accumulation of extracellular glucose indicates that this pathway did not predominate in this cultivation.

The DCW increased continuously and reached 27.7 g/L_Culture_ after 17 h, after which it stagnated. This corresponded to an OD_600_ of 60 and a growth rate of 0.22 h^− 1^. The vol. L-AI activity increased in parallel with the cell growth, reaching 54.1 ± 4.09 µkat_Gal, 65 °C_/L_Culture_ after 17 h. The corresponding L-AI activities were 2.0 ± 0.1 µkat_Gal, 65 °C_/g_DCW_ and 0.9 ± 0.1 µkat_Gal, 65 °C_/L_Culture_/OD_600_. The increase in activity, reaching a maximum at the end of the exponential phase, is consistent with the reported expression profile of the P_AprE_ promoter, which is most active during the transition from the exponential to the stationary phase [51].

**Fig. 4:**
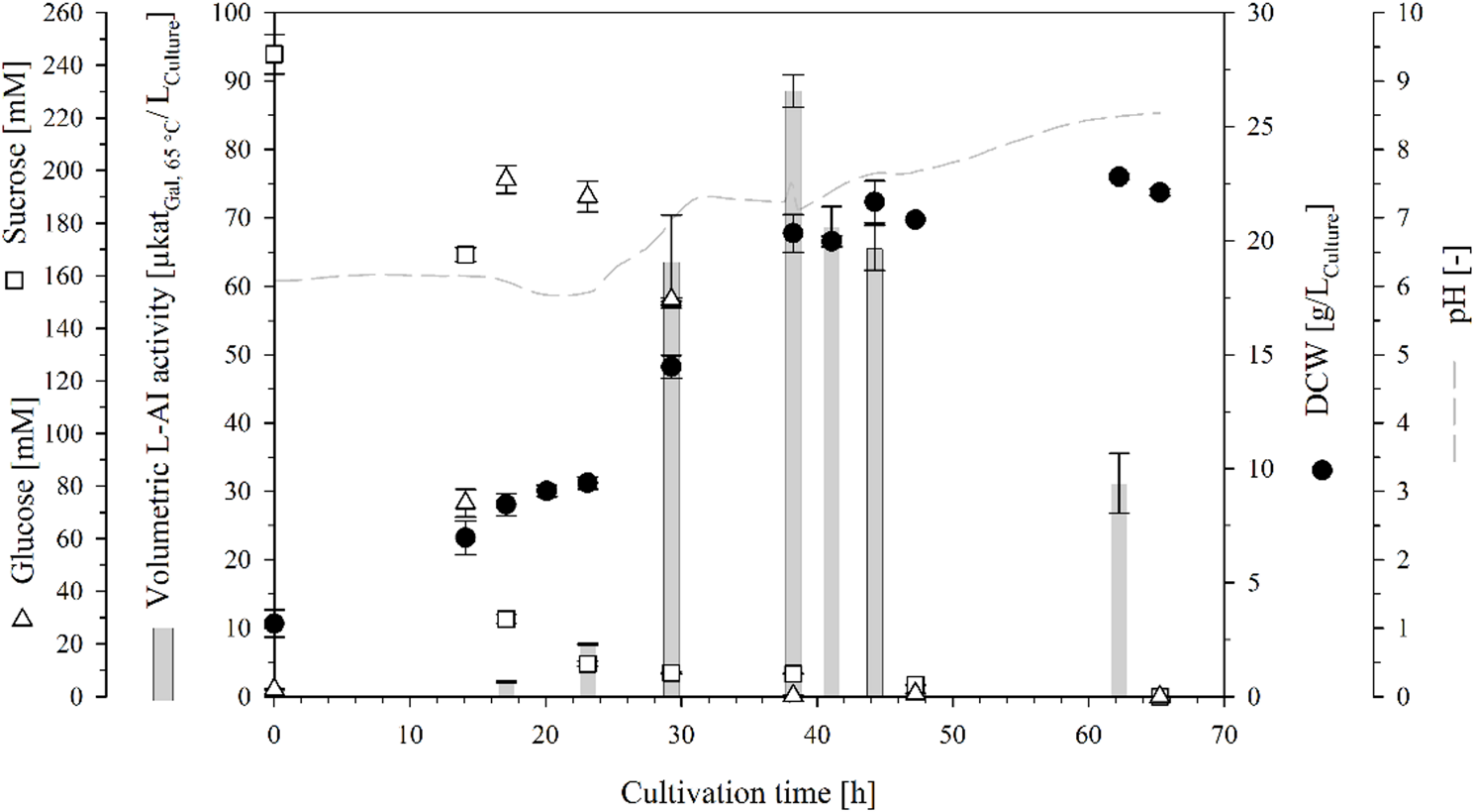
Bioreactor cultivation of *Bs*007∆*ss*-Ap for the intracellular production of L-AI-Lp. The batch cultivation was done with a working volume of 0.8 L at 30 °C, with unregulated pH and DO ≤ 5%

Although the shake flask and bioreactor cultivation of *Bs*007∆*ss*-Ap reached a similar OD_600_ of 60, the vol. L-AI activity of the bioreactor cultivation was 2.7-fold lower. This difference may be attributed to the different cultivation conditions, particularly the constant pH of 7 and DO > 30% in the bioreactor. Therefore, an additional bioreactor cultivation of *Bs*007∆*ss*-Ap was done with unregulated pH and DO ≤ 5% to simulate the conditions of shake flask cultivation. As expected, the cell growth was slower under these conditions, reaching a DCW of 22.8 g/L_Culture_ (OD_600_ = 45) after 62 h, with a growth rate of 0.04 h^− 1^ (Fig. 4). The 5.5-fold lower growth rate was probably due to the reduced oxygen availability, which was also observed in the study by Hu et al. [52]. The pH of the fermentation broth increased to 8.5 after 65 h, which was similar to the results of the shake flask cultivations. Furthermore, the vol. L-AI activity increased to 88.6 ± 2.4 µkat_Gal, 65 °C_/L_Culture_ after 38 h, corresponding to L-AI activities of 4.4 ± 0.1 µkat_Gal, 65 °C_/g_DCW_ and 1.8 ± 0.1 µkat_Gal, 65 °C_/L_Culture_/OD_600_. In summary, the vol. L-AI activity increased 1.6-fold under unregulated pH and low-oxygen conditions compared with the bioreactor cultivation at a constant pH of 7 and DO > 30%.

Cell-free extracts of samples taken during the two bioreactor cultivations were analyzed by SDS‒PAGE. A protein band of approximately 50 kDa, which is consistent with the theoretical monomeric molecular weight of L-AI-Lp (53.8 kDa, calculated from the amino acid sequence), was observed with increasing intensity throughout the cultivations (Additional file 1: Figure S4, S5).

### Partial purification of L-AI-Lp

**Fig. 5:**
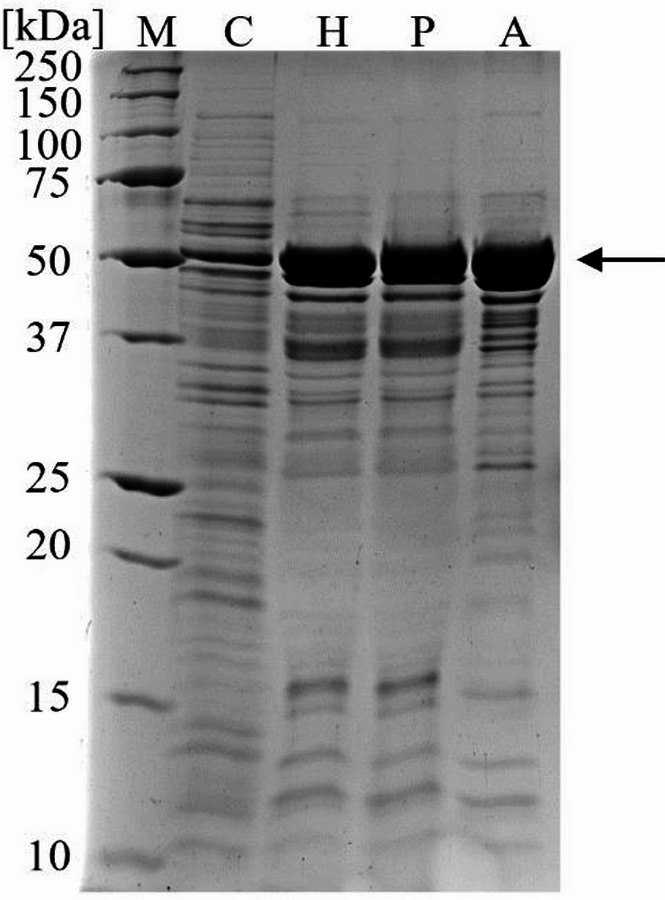
SDS‒PAGE analysis of partial purification of L-AI-Lp by heat treatment, polyethyleneimine, and ammonium sulfate precipitation.* M* protein standard,* C* cell-free extract after cell disruption,* H* heat treatment,* P* polyethyleneimine precipitation,* A* ammonium sulfate precipitation. The arrow indicates the theoretical molecular weight of an L-AI-Lp monomer

After the bioreactor cultivation of *Bs*007∆*ss*-Ap at an unregulated pH and DO ≤ 5% to produce L-AI-Lp intracellularly, 20.5 g of wet cells were disrupted using lysozyme, yielding a total L-AI activity of approximately 10 µkat_Gal, 65 °C,_ and a specific L-AI activity of 16.5 nkat_Gal, 65 °C_/mg_Protein_ (Table 2). Subsequently, heat-labile proteins of *B. subtilis* were denatured by heat treatment at 65 °C since L-AI-Lp showed a high thermostability at this temperature [8]. The specific L-AI activity increased 6.6-fold to 108.6 nkat_Gal, 65 °C_/mg_Protein,_ with a yield of 99%. This was followed by polyethyleneimine precipitation to remove negatively charged macromolecules, particularly nucleic acids [53]. In addition, fractionated ammonium sulfate precipitation was done to further purify and concentrate the L-AI-Lp. After both precipitation steps, the purification factor increased to 7.8, resulting in a specific L-AI activity of 128.2 nkat_Gal, 65 °C_/mg_Protein_ and a yield of 84%. The SDS‒PAGE analysis confirmed the purification of L-AI-Lp, showing a protein band with increasing intensity over the purification at approximately 50 kDa, which corresponds to the theoretical molecular weight of a monomer of L-AI-Lp (Fig. 5).

**Table 2:** Partial purification of L-AI-Lp after the bioreactor cultivation of *Bs*007∆*ss*-Ap

Sample	Volume [mL]	Total protein [mg]	Total activity [nkat_Gal, 65 °C_]	Specific activity [nkat_Gal, 65 °C_/mg]	Yield [%]	Purification [*n*-fold]
Cell-free extract	51.2	610.3	10,078	16.5	100.0	1.0
Heat treatment	44.8	92.0	9989	108.6	99.1	6.6
Polyethyleneimine precipitation	43.0	91.5	9877	108.0	98.0	6.5
Ammonium sulfate precipitation	6.5	65.7	8421	128.2	83.6	7.8

### Influence of Co^2+^ ions and glycerol on the storage stability of L-AI-Lp

After the partial purification of L-AI-Lp, the influences of Co^2+^ ions and glycerol on its storage stability in MES buffer at 4 °C and − 20 °C were investigated. Co^2+^ ions were tested because the L-AI-Lp showed maximal activity in their presence [8]. Previous studies have also shown that Co^2+^ ions can increase the thermostability of L-AIs [54, 55]. In addition, glycerol has been investigated because it is commonly used to increase protein stability in aqueous solutions [56].

**Table 3:** Storage stability of L-AI-Lp

Additive	Time [months]	Residual activity [%]	
		4 °C	-20 °C
None^a^	1	97 ± 2	104 ± 4
	2	82 ± 2	91 ± 4
	3	80 ± 1	96 ± 3
	6	64 ± 4	84 ± 2
Co^2+^ ions^b^	1	96 ± 5	97 ± 4
	2	99 ± 5	99 ± 4
	3	91 ± 2	92 ± 5
	6	86 ± 6	80 ± 2
Glycerol^c^	1	101 ± 5	95 ± 5
	2	99 ± 5	95 ± 6
	3	100 ± 5	97 ± 2
	6	87 ± 5	93 ± 5

Residual L-AI activity after incubation for up to 6 months at 4 °C or -20 °C in 25 mM MES buffer (pH 6.5) without additives (none), with 1 mM CoCl_2_ (Co^2+^ ions), or with 40% (v/v) glycerol (glycerol). ^a^100% = 286.9 ± 3.3 nkat_Gal, 65 °C_/mL. ^b^100% = 278.0 ± 16.6 nkat_Gal, 65 °C_/mL. ^c^100% = 226.0 ± 13.0 nkat_Gal, 65 °C_/mL

The relative activity of L-AI-Lp decreased to 64 ± 4% after 6 months of storage at 4 °C in MES buffer (Table 3). The addition of 1 mM Co^2+^ ions or 40% (v/v) glycerol significantly increased the stability of L-AI-Lp compared with the no-additive sample (*p* < 0.01 for each comparison), retaining 86 ± 6% and 87 ± 5% of the activity, respectively. Storage at -20 °C resulted in higher L-AI stability, with a relative activity of 84 ± 2% after 6 months in MES buffer. Under these conditions, Co^2+^ ions did not increase the stability (80 ± 2%), whereas glycerol increased it slightly to a relative activity of 93 ± 5% after 6 months.

## Discussion

The recombinant production of the previously discovered L-AI from *Lentilactobacillus parakefiri* in *B. subtilis* was systematically investigated in this study. Recombinant *B. subtilis* strains in combination with different promoters were compared for the intracellular production of L-AI-Lp. Two constitutive (P_AprE_, P_SecA_) and two inducible (P_SacB_, P_ManP_) promoters were investigated using *B. subtilis* 007 as the expression host to compare complementary expression strategies. The inducer-independent promoter P_AprE_ is most active during the transition from exponential to stationary phase [51], which supports L-AI-Lp production after biomass accumulation. In contrast, P_SecA_ is most active in the exponential phase and, like P_AprE_, does not require an inducer [38]. Furthermore, P_SacB_ and P_ManP_ are induced by sucrose and mannose, respectively, which avoids the use of synthetic inducers (e.g., isopropyl *β-*D-1-thiogalactopyranoside (IPTG)) [39, 40].

P_AprE_ yielded the highest vol. L-AI activity (69.2 µkat_Gal, 65 °C_/L_Culture_) among the investigated promoters, which was ≥ 1.4-fold higher than the activities obtained with the other promoters. The P_AprE_ promoter natively regulates the expression of *aprE* encoding the alkaline serine protease subtilisin E (EC 3.4.21.62), which contributes to ≈ 30% of the total extracellular protease activity in *B. subtilis* BG16, as reported by Yang et al. [57]. P_AprE_ has been widely used for the recombinant production of several enzymes in *B. subtilis* because of its expression strength [33, 58] and showed higher enzyme yields compared to other promoters [23]. Nevertheless, the other promoters investigated in this study, P_SecA_, P_SacB_, and P_ManP,_ also showed high production yields in previous studies [41, 59, 60]. These results emphasize the importance of promoter selection in optimizing recombinant L-AI-Lp production in *B. subtilis*. However, variations in plasmid copy number may also have contributed to the observed differences in L-AI-Lp yield and could be quantified using qPCR. Furthermore, future studies could combine the regulatory characteristics of the promoters with process parameters such as DO. P_AprE_, for example, could be used in a two-stage cultivation in which cells are first grown to a defined density and *L-AI-Lp* expression is then enhanced under low-DO conditions that promote the transition to the stationary phase.

Excessive foaming during recombinant protein production in bioreactors can lead to a loss of sterility and reduced product yield [61]. Therefore, the surfactin-deficient *B. subtilis* strains 007 ∆*sfp* ∆*sigF* and 168 were investigated as expression hosts for the recombinant production of L-AI-Lp. *B*. *subtilis* 007 ∆*sfp* ∆*sigF* lacks *sfp*, and *B*. *subtilis* 168 has a frameshift mutation in *sfp* [62]. The *sfp* gene encodes Sfp, a phosphopantetheinyl transferase that activates the peptidyl carrier protein domains of a surfactin synthetase [63]. Less foaming was observed in the cultivation of these strains than in the cultivation of the surfactin-producing *B. subtilis* 007 strains. This is relevant to the process because excessive foaming can result in a loss of culture volume, thereby reducing the total yield of L-AI-Lp.

The shake flask cultivation of *Bs*007∆*ss*-Ap yielded the highest vol. L-AI activity of 147.7 µkat_Gal, 65 °C_/L_Culture_, which was 2.1-fold higher than that with *Bs*007-Ap. In both cultivations, the maxima of L-AI activity were observed at the transition to the stationary phase, which is consistent with the reported expression profile of the P_AprE_ promoter [51]. However, the highest activity with *Bs*007∆*ss*-Ap was measured after 39 h of cultivation, whereas that for *Bs*007-Ap was observed after ≈ 20 h. This is consistent with a longer exponential phase of *Bs*007∆*ss*-Ap. One possible explanation is the absence of surfactin in the cultivation of *Bs*007∆*ss*-Ap. Surfactin can initiate KinC–Spo0A–dependent expression of genes for matrix production and biofilm formation during the transition phase [64]. The absence of surfactin could have delayed the expression of these genes and the onset of stationary phase, thereby shifting the time point of the highest measured L-AI activity.

Furthermore, previous studies have shown that the deletion of *sigF* can increase recombinant enzyme production [43, 65]. For instance, the deletion of *sigF* in *B. licheniformis* and *B*. *amyloliquefaciens* in the studies by Zhou et al. and Zhang et al., respectively, increased the extracellular subtilisin E production by 20–25% compared to the respective wild-type strain [43, 65]. Furthermore, Wang et al. reported that deletion of sporulation-related genes (*spo0A*, *spoIIIE*, and *spoIVB*) in *B. subtilis* 168 increased the production of a heterologous *β*-galactosidase and an *α*-amylase [30]. Transcriptome analyses revealed that genes involved in DNA replication, mRNA transcription, and protein folding were upregulated in nonsporulating *B. subtilis* strains, which may have contributed to the increased production of heterologous enzymes [30]. Although it has been reported that deletion of *spo0A* can increase heterologous enzyme production, Spo0A is a master regulator whose inactivation can cause pleiotropic effects [66]. Therefore, *sigF* was deleted instead of *spo0A* in the present study.

The strain *Bs*007∆*ss*-Ap showed the highest production of L-AI-Lp in shake flask cultivation, with a vol. L-AI activity of 147.7 µkat_Gal, 65 °C_/L_Culture_. However, the L-AI activity decreased to 54.1 µkat_Gal, 65 °C_/L_Culture_ in a bioreactor cultivation with a constant pH of 7.0 and DO > 30%. This decrease may be caused by the controlled cultivation parameters, particularly the constant pH and DO > 30%. Therefore, a bioreactor cultivation with unregulated pH and DO ≤ 5% was done to simulate the conditions of the shake flask cultivation. Under these conditions, the vol. L-AI activity increased to 88.6 µkat_Gal, 65 °C_/L_Culture_. This result is consistent with previous studies that have demonstrated the influence of DO and pH on the production of recombinant enzymes in *B*. *subtilis* [24, 25, 67]. Therefore, future studies should measure DO and the oxygen transfer rate during the shake flask cultivation of *Bs*007∆*ss*-Ap and evaluate their effects on L-AI-Lp production in bioreactor cultivations.

In a previous study, the L-AI-Lp was recombinantly produced in *E. coli* BL21(DE3) in a bioreactor with a production yield of 27.7 µkat_Gal, 65 °C_/L_Culture_, which is 3.2-fold lower than the yield in this study [8]. Furthermore, the L-AI yield obtained in the present study was higher than that of previous studies investigating the recombinant production of L-AIs in prokaryotes with a QPS status [13, 14]. Salonen et al. investigated the intracellular production of L-AI from *Bifidobacterium longum* in *Lactococcus lactis* during shake flask cultivations, resulting in an L-AI yield of 13.3 µkat_D−Gal_, _45 °C_/L_Culture_ [13]. Moreover, Staudigl et al. cultivated recombinant *Lactobacillus plantarum* strains in shake flasks for the intracellular production of L-AI from *Lactobacillus reuteri*, which yielded 0.5 µkat_D−Gal, 65 °C_/L_Culture_ [14]. Other studies have focused on the intracellular production of L-AIs in *B. subtilis* [15, 68–70]. However, a direct comparison of the production yields is not possible because quantitative data on vol. L-AI activities are missing.

Many studies have reported the purification of L-AIs using chromatography-based methods, such as immobilized metal affinity chromatography and anion exchange chromatography [8, 14, 71, 72]. In the study by Weber et al., the L-AI-Lp was recombinantly produced in *E*. *coli* and purified 2.9-fold by heat treatment followed by anion exchange chromatography. This resulted in a yield of 71% and a specific L-AI activity of 99.6 nkat_Gal, 65 °C_/mg_Protein_, which was determined under assay conditions similar to those used in the present study [8]. However, chromatographic methods are limited by the high cost of resins on an industrial scale [73]. Therefore, in the present study, the L-AI-Lp was partially purified by heat treatment, polyethyleneimine precipitation, and ammonium sulfate precipitation. This approach resulted in a 7.8-fold increase in specific L-AI activity with 128.2 nkat_Gal, 65 °C_/mg_Protein_ and a yield of 84%. Compared with the method reported by Weber et al. [8], the current approach showed a higher specific L-AI activity and yield, demonstrating its potential as a scalable alternative to chromatographic purification. Nevertheless, the purity of L-AI-Lp could be further increased by additional purification steps (e.g., cross-flow filtration).

The influences of glycerol and Co^2+^ ions on the storage stability of L-AI-Lp were investigated. Glycerol increased the stability of the L-AI, resulting in residual activities of 87 ± 5% at 4 °C and 80 ± 2% at -20 °C after 6 months of storage. The stability of enzymes in aqueous solutions is often increased by glycerol [74]. The latter reduces molecular flexibility, thereby reducing protein unfolding and aggregation [56]. It also stabilizes proteins by reducing the formation of ice crystals [75]. The influence of Co^2+^ ions on the storage stability was also investigated because other studies have shown that Co^2+^ ions increase the thermostability of some L-AIs [54, 55]. At -20 °C, Co^2+^ ions did not influence the storage stability, with a relative activity of 80 ± 2% after 6 months. However, at 4 °C, Co^2+^ ions increased the stability by approximately 1.3-fold, resulting in a residual activity of 86 ± 6% after 6 months. These results are comparable to those of Weber et al., who investigated the storage stability of L-AI-Lp produced in *E*. *coli* and stored at 4 °C and − 20 °C in MES buffer containing 1 mM CoCl_2_ [9]. The L-AI-Lp retained 75 ± 4% and 97 ± 4% of its activity after 6 months at 4 °C and − 20 °C, respectively [9]. Future experiments could analyze the combined influence of glycerol and Co^2+^ ions on L-AI-Lp stability, as the present study only investigated their individual influence.

## Conclusions

This study reports for the first time the recombinant production of L-AI from *Lentilactobacillus parakefiri* DSM 10551 in a microbial species with a QPS status, using the nonsporulating and surfactin-deficient strain *B. subtilis* 007 ∆*sfp* ∆*sigF*. Systematic investigation of promoters, *B. subtilis* strains, and cultivation parameters (pH and DO) increased L-AI production and provides a valuable basis for further process optimization. In addition, the nonchromatographic purification strategy for L-AI-Lp could be used as a cost-effective method, particularly on an industrial scale. The results of this study represent a step toward the potential industrial application of L-AI-Lp. Furthermore, this study highlights the potential of *B. subtilis* 007 ∆*sfp* ∆*sigF* for producing L-AIs, surpassing the performance of previously used expression hosts from the order *Lactobacillales*. Future experiments should replace plasmid-based expression with antibiotic-free, multi-copy chromosomal integration of the L-AI-Lp gene in *B. subtilis* 007 ∆*sfp* ∆*sigF*.

## Supplementary Information

Below is the link to the electronic supplementary material.

Supplementary Material 1.

## Data Availability

All data generated or analyzed during this study are included in this published article and its supplementary information file.
