# Supplementary material for: Recombinant production of acidophilic L-arabinose isomerase from Lentilactobacillus parakefiri in Bacillus subtilis
Source: Microb Cell Fact. 2025 Dec 22;24:251. doi: 10.1186/s12934-025-02900-z (PMC12752274; doi:10.1186/s12934-025-02900-z)
Supplement: Supplementary file 1 — Supplementary Material 1. [file 12934_2025_2900_MOESM1_ESM.docx]

**Additional file 1**

**Recombinant production of acidophilic L-arabinose isomerase from *Lentilactobacillus parakefiri* in *Bacillus subtilis***

Nathanael Weber^1^, Sebastian Götz^1,2^, Jana Senger^1^, Sabine Lutz-Wahl^1^ and Lutz Fischer^1^*

^1^University of Hohenheim, Institute of Food Science and Biotechnology, Department of Biotechnology and Enzyme Science, Garbenstr. 25, 70599 Stuttgart, Germany

^2^Present address: Biberach University of Applied Sciences, Institute for Applied Biotechnology, Karlstr. 11, 88400 Biberach, Germany

*Corresponding author:

**Table S1. Primer sequences used in this study.**

| **Primer** | **Sequence (5' -> 3')** |
| --- | --- |
| P1 | cgactagtatgttacaagtacctgattatg |
| P2 | gctcgagttattacttgatgtcgttaaatg |
| P3 | cggaattcccatcacatatacctg |
| P4 | ggactagtcatgtctccttttttatg |
| P5 | ctcaggaattcttctgtacaccaatgtcatg |
| P6 | gtactagtttttgcctcctaacttaagaataag |
| P7 | ctgaattccgtaaattccgtgagcagg |
| P8 | cgcgcgccgtctctctagtttataacgctcctctatcatcacac |
| P9 | gacatttaacgacatcaagtaataaacataaaaaaccggcc |
| P10 | cataatcaggtacttgtaacatttataacgctcctctatcatc |
| P11 | gatgatagaggagcgttataaatgttacaagtacctgattatg |
| P12 | ccaaggccggttttttatgtttattacttgatgtcgttaaatgtc |
| P13 | aaggccaacgaggccgcatgagccttggaattgac |
| P14 | aaggccttcagggcctctccttaattacaaagcgc |
| P15 | aaggccctgaaggccctagtctgcagtgcaggctag |
| P16 | aaggccttattggcccaccgttatgcctccgccg |
| P17 | aaggccaacgaggccctgtcagatgtgctacaatgac |
| P18 | aaggccttcagggcccattctagatcctccgtctgc |
| P19 | aaggccctgaaggccgctcatcaacagcttgacac |
| P20 | aaggccttattggccgaagatcgccattgaacagc |

**Table S2. Plasmids used in this study.**

| **Plasmid** | **Description** | **Reference** |
| --- | --- | --- |
| pLF_P_AprE__AprE | Negative control | [1] |
| pLF_P_AprE__L-AI-Lp | Expression of L-AI-Lp | This work |
| pLF_P_SecA__L-AI-Lp | Expression of L-AI-Lp | This work |
| pLF_P_SacB__L-AI-Lp | Expression of L-AI-Lp | This work |
| pLF_P_ManP__L-AI-Lp | Expression of L-AI-Lp | This work |
| pJOE8999 | CRISPR/Cas9 plasmid for *B. subtilis* | [2] |
| pJOE8999_sgSigF | CRISPR/Cas9 plasmid with integrated sgRNA for *sigF* | [3] |
| pJOE8999_sgSfp | CRISPR/Cas9 plasmid with integrated sgRNA for *sfp* | [3] |
| pJOE8999_DsigF | CRISPR/Cas9 plasmid for deletion of *sigF* | This work |
| pJOE8999_Dsfp | CRISPR/Cas9 plasmid for deletion of *sfp* | This work |

**Table S3. Recombinant B. subtilis strains used in this study.**

| **Designation** | **Strain** | **Plasmid** | **Promoter** |
| --- | --- | --- | --- |
| *Bs*007-Ap | *B*. *subtilis* 007 | pLF_P_AprE__L-AI-Lp | P_AprE_ |
| *Bs*007-Se | *B*. *subtilis* 007 | pLF_P_SecA__L-AI-Lp | P_SecA_ |
| *Bs*007-Sa | *B*. *subtilis* 007 | pLF_P_SacB__L-AI-Lp | P_SacB_ |
| *Bs*007-Ma | *B*. *subtilis* 007 | pLF_P_ManP__L-AI-Lp | P_ManP_ |
| *Bs*007∆*ss*-Ap | *B*. *subtilis* 007 ∆*sfp* ∆*sigF* | pLF_P_AprE__L-AI-Lp | P_AprE_ |
| *Bs*007∆*ss*-nc | *B*. *subtilis* 007 ∆*sfp* ∆*sigF* | pLF_P_AprE__AprE | P_AprE_ |
| *Bs*168-Ap | *B*. *subtilis* 168 | pLF_P_AprE__L-AI-Lp | P_AprE_ |
| *Bs*168-nc | *B*. *subtilis* 168 | pLF_P_AprE__AprE | P_AprE_ |


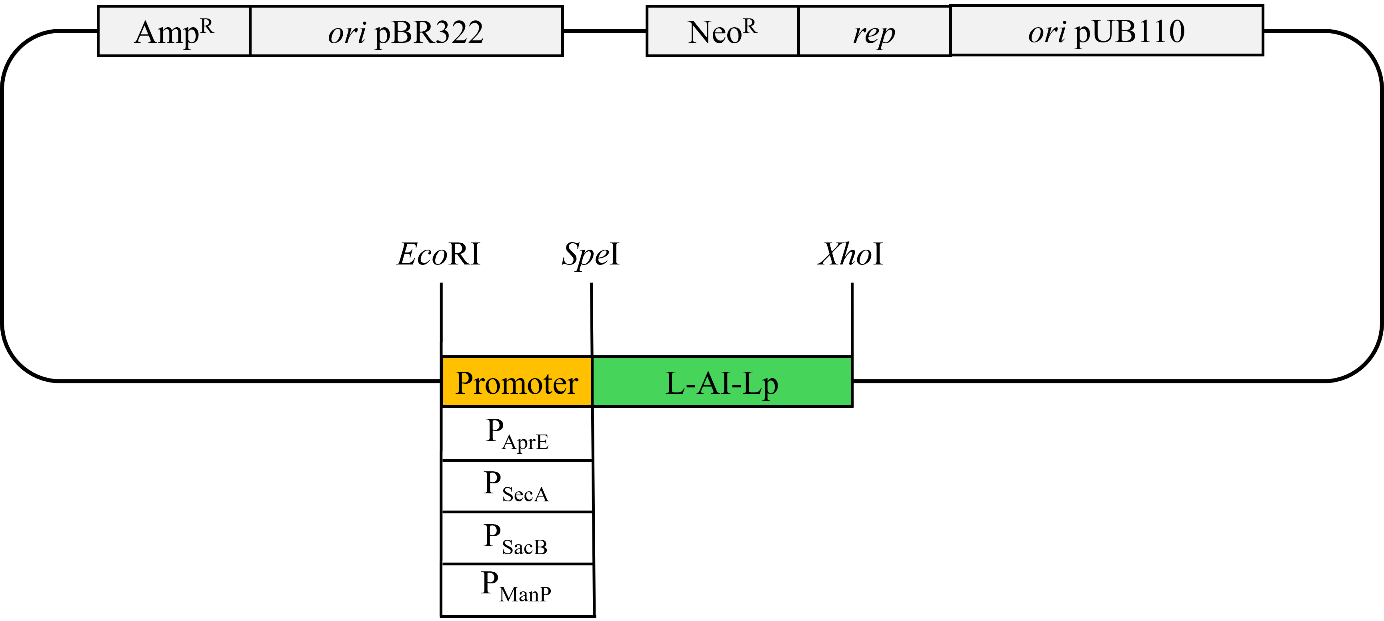


**Figure S1.** Schematic overview of the constructed expression plasmids. The plasmids were constructed based on a modular expression cassette previously integrated into the pLF shuttle vector [1]. Amp^R^: ampicillin resistance. ori pBR322: origin of replication derived from pBR322. Neo^R^: neomycin resistance. rep: replication initiation gene from pUB110. ori pUB110: origin of replication derived from pUB110. The figure is based on Senger et al. with modifications [1].

*
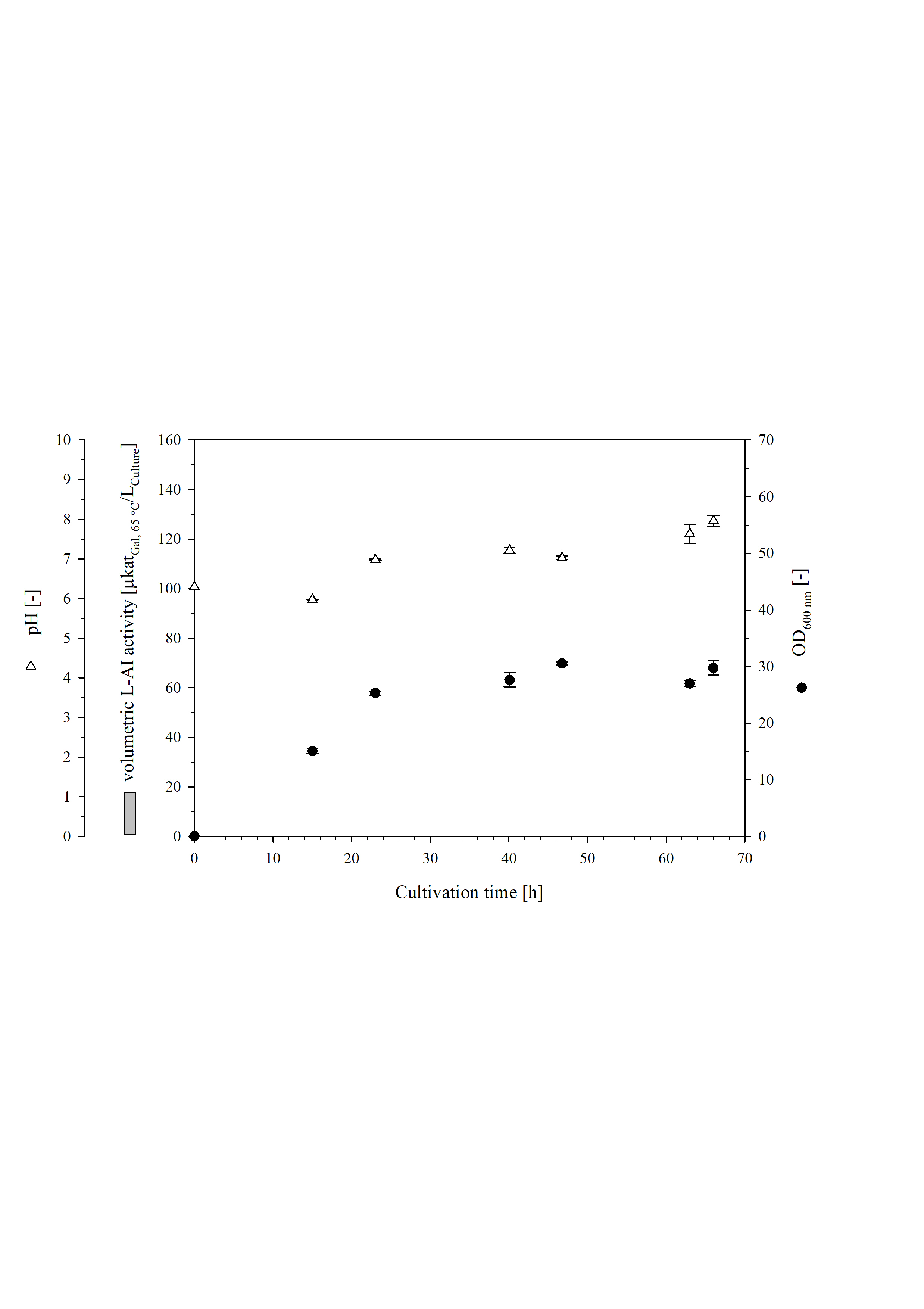
*

**Figure S2.** Shake flask cultivation of Bs007∆ss-nc. The batch cultivation was done with a working volume of 150 mL at 30 °C.


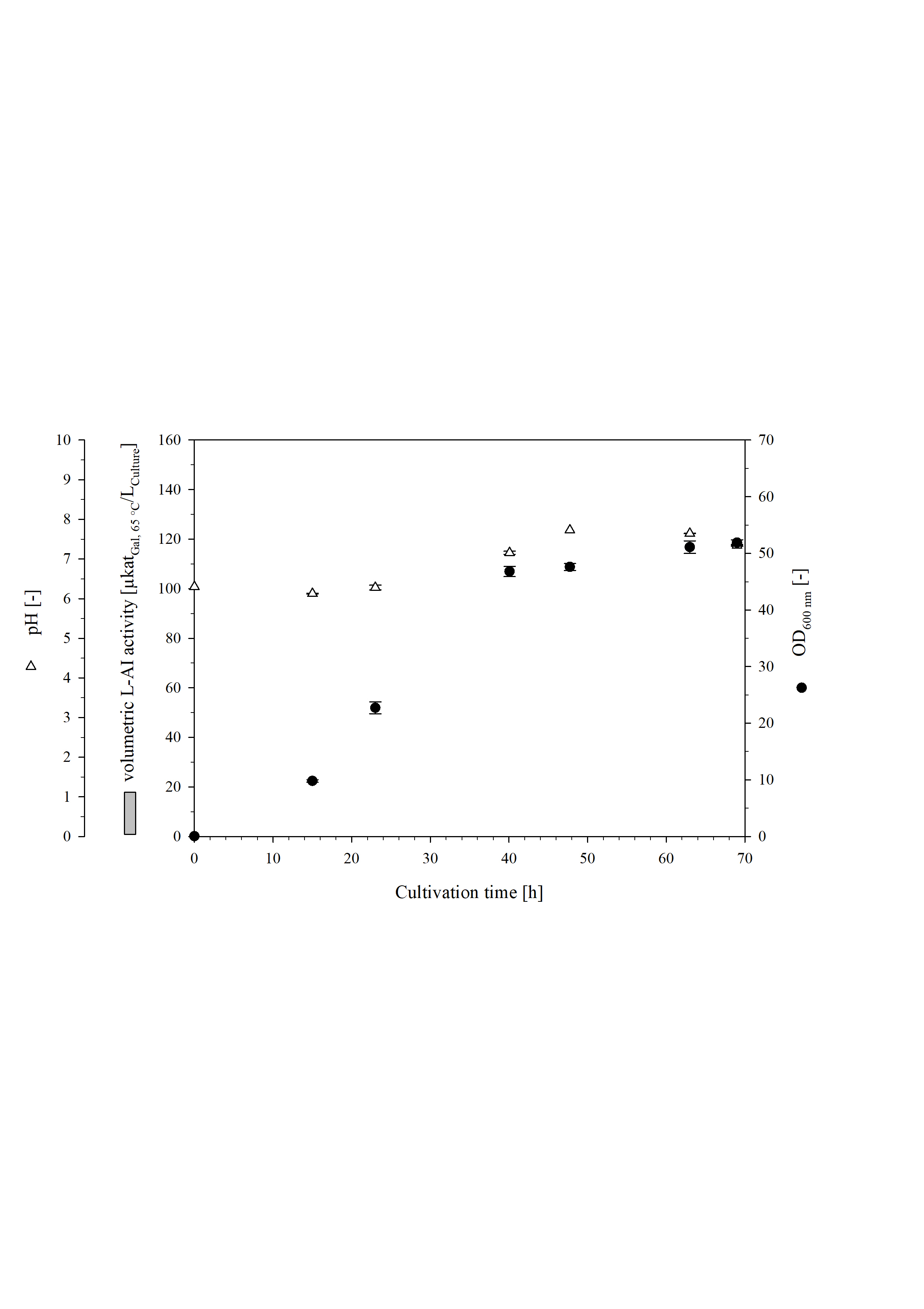


**Figure S3.** Shake flask cultivation of Bs168-nc. The batch cultivation was done with a working volume of 150 mL at 30 °C.


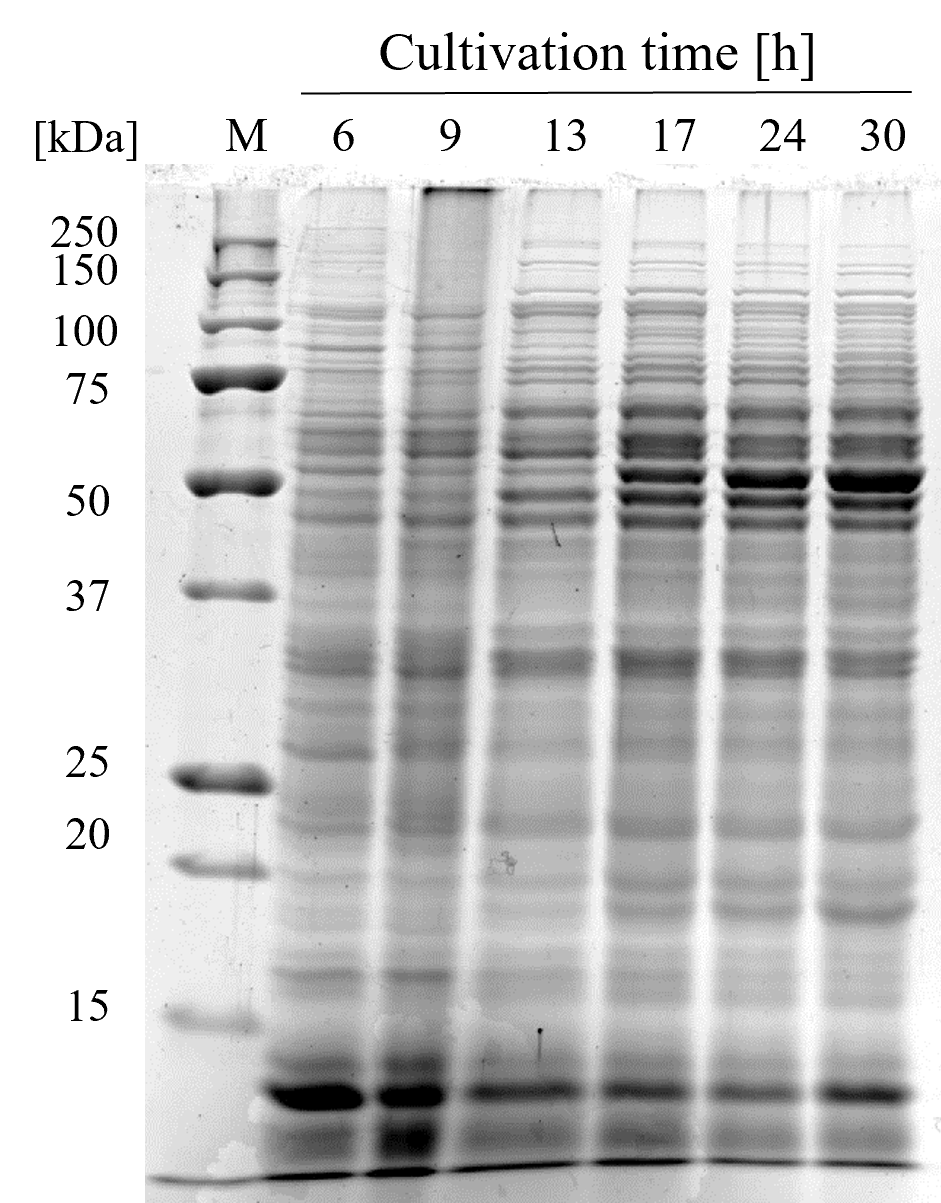


**Figure S4.** SDS-PAGE analysis of the cell-free extracts from bioreactor cultivation of Bs007∆ss-Ap with pH 7 and DO > 30%. M: precision Plus Protein™ unstained protein standard.


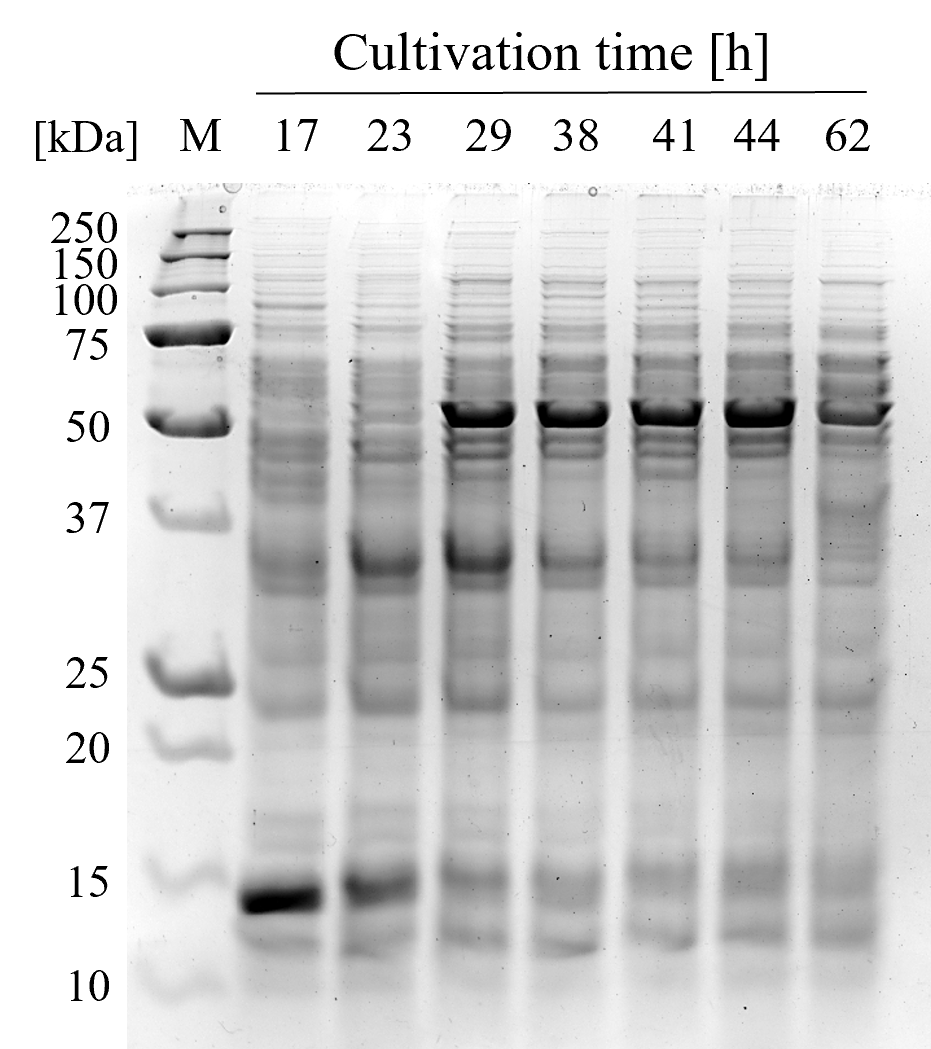


**Figure S5.** SDS-PAGE analysis of the cell-free extracts from bioreactor cultivation of Bs007∆ss-Ap with unregulated pH and DO ≤ 5%. M: precision Plus Protein™ unstained protein standard.

**Reference**

1. Senger J, Seitl I, Pross E, Fischer L. Secretion of the cytoplasmic and high molecular weight *β*-galactosidase of *Paenibacillus wynnii* with *Bacillus subtilis*. Microb Cell Fact. 2024;23:170.

2. Altenbuchner J. Editing of the *Bacillus subtilis* genome by the CRISPR-Cas9 system. Appl Environ Microbiol. 2016;82(17):5421–7.

3. Senger J, Keutgen M, Roth N, Seitl I, Fischer L. Toward food-grade production of the *Bacteroides helcogenes* protein-glutamine glutaminase with an optimized *Bacillus subtilis* strain. Research Square 2025; Preprint (Version 1).
